# Antiviral activity of glucosylceramide synthase inhibitors in alphavirus infection of the central nervous system

**DOI:** 10.1093/braincomms/fcad086

**Published:** 2023-03-25

**Authors:** Roy Avraham, Sharon Melamed, Hagit Achdout, Noam Erez, Ofir Israeli, Moria Barlev-Gross, Metsada Pasmanik-Chor, Nir Paran, Tomer Israely, Einat B Vitner

**Affiliations:** Department of Infectious Diseases, Israel Institute for Biological Research, 7410001 Ness-Ziona, Israel; Department of Infectious Diseases, Israel Institute for Biological Research, 7410001 Ness-Ziona, Israel; Department of Infectious Diseases, Israel Institute for Biological Research, 7410001 Ness-Ziona, Israel; Department of Infectious Diseases, Israel Institute for Biological Research, 7410001 Ness-Ziona, Israel; Department of Biochemistry and Molecular Genetics, Israel Institute for Biological Research, 7410001 Ness-Ziona, Israel; Department of Infectious Diseases, Israel Institute for Biological Research, 7410001 Ness-Ziona, Israel; Bioinformatics Unit, George S. Wise Faculty of Life Science, Tel Aviv University, 6997801 Tel Aviv, Israel; Department of Infectious Diseases, Israel Institute for Biological Research, 7410001 Ness-Ziona, Israel; Department of Infectious Diseases, Israel Institute for Biological Research, 7410001 Ness-Ziona, Israel; Department of Infectious Diseases, Israel Institute for Biological Research, 7410001 Ness-Ziona, Israel

**Keywords:** encephalitis, antiviral drug, sphingolipids, alphaviruses, glucosylceramide

## Abstract

Virus-induced CNS diseases impose a considerable human health burden worldwide. For many viral CNS infections, neither antiviral drugs nor vaccines are available. In this study, we examined whether the synthesis of glycosphingolipids, major membrane lipid constituents, could be used to establish an antiviral therapeutic target. We found that neuroinvasive Sindbis virus altered the sphingolipid levels early after infection *in vitro* and increased the levels of gangliosides GA1 and GM1 in the sera of infected mice. The alteration in the sphingolipid levels appears to play a role in neuroinvasive Sindbis virus replication, as treating infected cells with UDP-glucose ceramide glucosyltransferase (UGCG) inhibitors reduced the replication rate. Moreover, the UGCG inhibitor GZ-161 increased the survival rates of Sindbis-infected mice, most likely by reducing the detrimental immune response activated by sphingolipids in the brains of Sindbis virus-infected mice. These findings suggest a role for glycosphingolipids in the host immune response against neuroinvasive Sindbis virus and suggest that UGCG inhibitors should be further examined as antiviral therapeutics for viral infections of the CNS.

## Teaser

Avraham *et al*. reported that inhibitors of glucosylceramide synthase have antiviral activity and can enhance the survival rate of mice with viral encephalitis. Treatment with the inhibitor reduced inflammation in the brain, suggesting a new role of glucosylceramide synthase as an immunosuppressive drug.

## Graphical Abstract

**Graphical abstract:**
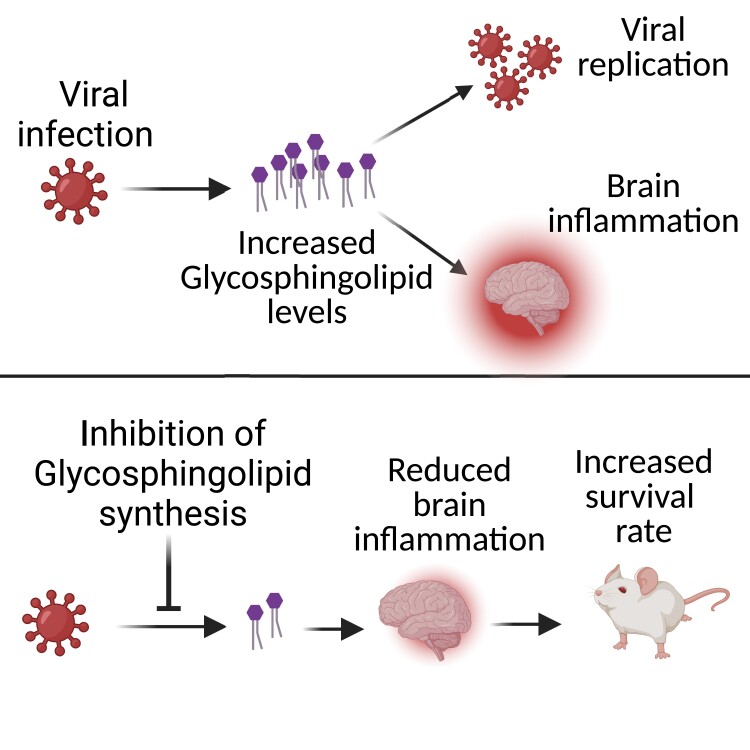

## Introduction

Viral encephalomyelitis can be a devastating disease to the infected individuals and society as a whole due to long-term neurological sequelae such as paralysis and cognitive deficits.^1^ Arthropod-borne (arbo) viruses are major causes of encephalomyelitis with widespread seasonal outbreaks.^2^ Alphaviruses are mosquito-borne plus-strand-enveloped RNA viruses in the Togaviridae family that cause both encephalomyelitis (Venezuelan, western and eastern equine encephalitis viruses) and arthritis (Sindbis, Ross River, and Chikungunya viruses). Encephalitic alphaviruses are endemic in the Americas, while rapidly emerging arthritic alphaviruses that can also cause neurologic disease are now found worldwide.^1^

Currently, neither antiviral drugs nor vaccines are available for many viral infections of the central nervous system (CNS).^3^ As viruses rely on intracellular mechanisms, they must cross cell membranes during their life cycle and thus establish a dependency on processes involving membrane dynamics. Sphingolipids (SLs) are a major class of eukaryotic lipids. In addition to playing an essential role in the life cycle of viruses, SLs are regulators of the immune system, specifically the type 1 interferon (IFN) response,^4^ which might suppress viral infection.^5,6^

Glucosylceramide synthase (UGCG) (EC 2.4.1.80), also called UDP-glucose:ceramide glucosyltransferase, catalyzes the biosynthesis of glucosylceramide (GlcCer) from ceramide (Cer), which is the first step in the biosynthesis of gangliosides and other glycosphingolipids (GSLs).

A central role for glycosphingolipid synthesis in viral replication has been found in infections with several viruses; UGCG inhibitors and knockdown and/or knockout of UGCG inhibit influenza virus and severe acute respiratory syndrome coronavirus 2 (SARS-CoV-2).^7-9^ Moreover, the antiviral efficacy of iminosugar N-butyl-DNJ (NB-DNJ, miglustat) and DNJ derivatives in inhibiting the replication of viruses from different families has been demonstrated.^10-18^ Although these studies provide strong circumstantial evidence showing that the antiviral activity of iminosugars results from the inhibition of endoplasmic reticulum (ER) α-glucosidase activity,^19^ the ubiquity of d-glucose in metabolism suggests that other pathways may be equally affected by iminosugar treatment. Indeed, NB-DNJ has been approved for clinical use since 2002 as a second-line treatment for Gaucher disease (GD).^20^ In this context, NB-DNJ is used as an inhibitor of UGCG to reduce the production of glycosphingolipids that accumulate due to a deficiency in GlcCer degradation.^21^ The antiviral activity of specific UGCG inhibitors in viral infection of the CNS has not been examined *in vivo* or *in vitro*. Thus, in this study, we examined the therapeutic potential of specific UGCG inhibitors against a neurovirulent Sindbis virus strain with neuroinvasive properties (neuroinvasive Sindbis virus, SVNI).^22^ SVNI is a prototypical member of the alphavirus genus and has been used for many years to study the pathogenesis of acute viral encephalitis in mice.^23-25^ The following UGCG inhibitors were examined: (i) (1R,2R)-nonanoic acid[2-(2′,3′-dihydro-benzo [1,4] dioxin-6′-yl)-2-hydroxy-1-pyrrolidin-1-ylmethyl-ethyl]-amide-l-tartaric acid salt (Genz-123346), hereafter termed GZ-346, which is an analogue of the FDA-approved drug eliglustat (Cerdelga®) indicated for the long-term treatment of adult patients with type 1 Gaucher disease (GD)^26^ and (ii) (S)-quinuclidin-3-yl (2-(2-(4-fluorophenyl)thiazol-4-yl)propan-2-yl)carbamate (GENZ-667161), hereafter termed GZ-161, which is a specific inhibitor of UGCG that can access the CNS and has been demonstrated to effectively reduce glycosphingolipid synthesis.^27-29^ GZ-161 is an analogue of venglustat, which is currently in clinical trials for GD, Fabry disease, and Tay–Sachs disease and is in a pivotal phase 3 trial for autosomal dominant polycystic kidney disease.^27-29^ Although these specific UGCG inhibitors have been extensively studied for the treatment of lysosomal storage disorders, their antiviral therapeutic potential has not yet been explored.

We showed that SVNI alters the levels of SLs early after infection. This alteration appears to play a role in viral replication, as indicated by the finding that both UGCG inhibitors reduced the replication of SVNI *in vitro*. Moreover, GZ-161 significantly reduced certain host immune pathways in the brains of infected mice and increased the survival rate of SVNI-infected mice. Our data suggest that GSLs are involved in SVNI-induced pathology and that UGCG inhibitors have antiviral therapeutic potential against alphavirus-induced viral encephalitis.

## Materials and methods

### Cells

Vero (ATCC® CCL-81™) and Neuro-2a cells (ATCC® CCL-131™) were obtained from the American Type Culture Collection (Summit Pharmaceuticals International, Japan). The cells were used and maintained in Dulbecco’s modified Eagle’s medium (DMEM) supplemented with 10% heat-inactivated foetal calf serum (FCS), nonessential amino acids (NEAAs), 2 mM l-glutamine, 100 units/ml penicillin, 100 μg/ml streptomycin, and 1.25 units/ml nystatin at 37°C in an atmosphere consisting of 5% CO_2_/95% air.

### Viruses

The original strain of Sindbis virus (SV) was isolated in 1990 from mosquitoes in Israel. This strain was used as a source for variants with differing levels of neuroinvasiveness and virulence generated by serial passages of SV in suckling and weanling mouse brains. At passage 15, a neurovirulent variant was observed and designated SVN (neurovirulent). After seven more passages in weanling mouse brains, another variant was observed and designated SVNI (neuroinvasive). The SVNI strain used is both virulent and CNS-invasive.^13^

Recombinant TRNSV-Luc was kindly provided by Diane E. Griffin (Johns Hopkins Bloomberg School of Public Health, MD, USA).

Recombinant SIN-GFP was kindly provided by Nicolas Ruggli (N. Ruggli and C. M. Rice, unpublished data).^30^ WNV virus (NY-99, ATCC® VR-1507™) was used.

### UGCG inhibitors

Compounds GZ-161 (PubChem Identifier: 60199242; URL: https://pubchem.ncbi.nlm.nih.gov/compound/60199242) and GZ-346 (PubChem Identifier: 23652732; URL: https://pubchem.ncbi.nlm.nih.gov/compound/Genz-123346-free-base) were obtained from Sanofi. GZ-161 and GZ-346 were stored as 20 and 5 mM stock solutions in DMSO and PBS, respectively, at −20°C until use.

### Inhibition of SV in cell culture

Vero or N2a cells (3 × 10^4^ cells per well) were seeded in 96-well plates. After overnight incubation, the cells were treated in quadruplicate with serial dilutions (0.2–80 µM) of GZ-161 or GZ-346. One hour later, the cells were infected with TRNSV-Luc (MOI = 0.01). The infected cells were lysed 23 h later, and luciferase activity was measured using the Luciferase Assay System (Promega, Madison, WI, USA) with an Infinite 200 M Plex plate reader (TECAN).

The viability of uninfected cells was greater than 95%, as determined by an XTT assay (Merck; a colorimetric cell proliferation assay for the quantification of cell proliferation, cell viability and cytotoxicity). The half-maximal inhibitory concentration (IC50) values were evaluated with GraphPad Prism 6. The inhibition percentage was calculated as 1 minus the ratio of the plaque-forming units (PFUs) of treated cells to those of untreated cells.

### SVNI release assay

Vero cells were seeded at a density of 8 × 10^5^ cells per well in 6-well plates. After overnight incubation, the cells were treated in quadruplicate with 10 µM GZ-161, and 1 h later, the cells were infected with SVNI (MOI = 5). After 1 h of incubation with SVNI, the cells were washed five times with fresh media to wash away the virus that did not enter the cells. The supernatant was collected 24 h postinfection (hpi) for PFU quantification. For the quantification of PFUs, Vero cells were seeded at a density of 8 × 10^5^ cells per well in 6-well plates. After overnight incubation, cell monolayers were infected with serial dilutions of media, and 30–35 PFUs per well of live virus served as a control and were incubated for 48 h at 37°C. The inhibitory capacity of GZ-161 was then assessed by determining the number of plaques compared with that of untreated cells.

### Sphingolipid quantification

#### Sample preparation

Vero cells were seeded at a density of 1 × 10^6^ cells per 60-mm plate. After overnight incubation, the cells were treated in quadruplicate with 10 µM GZ-161 or GZ-346. One hour later, the cells were infected with SVNI (MOI = 5). At 3 hpi, the cells were washed three times with cold PBS and collected with a rubber policeman. Each cell pellet and each 50 µl serum sample were suspended in 100 μl of methanol:chloroform (1:1), and the samples were sent to The Metabolomics Innovation Centre (TMIC, Canada) for analysis. Each sample was mixed with 100 μl of a mixture of five deuterium-labeled sphingolipids, i.e. Cer (d18:1-d7/16:1), Cer (d18:1-d7/20:1), Cer (d18:1-d7/C24:1), SM (d18:1-d9/18:1) and C18:1 SM (d18:1-d9/22:1), all of which were acquired from Avanti Polar Lipids, Inc., as internal standards and 300 μl of methanol:chloroform (3:1) containing BHT as an antioxidant. The mixture was vortexed for 2 min at 3000 rpm and then ultrasonicated in an ice water bath for 5 min before clarification by centrifugation for 10 min at 21 000×g. The clarified supernatant was collected for LC-MRM/MS, and the protein pellet was used for protein quantitation via a standardized Bradford assay.

#### Calibration solutions and LC–MS

A mixed stock solution of 63 targeted sphingolipids (see Supplementary Table 1 for the full list, the concentration of each compound was 40 μM) was prepared using corresponding standard substances acquired from several commercial suppliers in methanol:chloroform (3:1) containing the same internal standards. This solution was serially diluted 1:4 (v/v) with the same solvent to obtain 10 calibration solutions. Ten-microlitre aliquots of the calibration solutions and the sample solutions were injected onto an LC column (C8, 2.1 × 50 mm, 1.7 μm) for UPLC–MS/MS on a Waters Acquity UPLC system coupled to a 4000 QTRAP mass spectrometer operated in the multiple reaction monitoring (MRM) mode with positive ion detection for sphingolipids and negative ion detection for sphingolipid phosphates. The mobile phase was 0.01% formic acid in water and acetonitrile–isopropanol (2:1) for binary solvent gradient elution (25–100% organic solvent in 12.5 min), which was followed by a 3-min column cleanup with 100% B and 4-min column equilibration with 25% B at 400 μl/min and 55°C. The ion transitions for MRM detection of each sphingolipid were optimized by a direct infusion of a standard solution to acquire two ion transitions per compound, with one ion transition as the quantifier and the other as the qualifier. The UPLC-MRM/MS data files were recorded using Sciex Analyst 1.6 software and processed using Sciex MultiQuant 2.0 software. Linear regression calibration curves of individual sphingolipids were constructed based on internal-standard calibration using the data acquired from injections of the calibration solutions in an appropriate concentration range for each lipid. The concentrations of the sphingolipids detected in each sample were calculated by interpolating the calibration curves of individual sphingolipids with the analyte-to-internal standard peak area ratios measured from injections of the sample solution. For the sphingolipids detected without the standard substances available, their concentrations were estimated using the calibration curves from one of the homologs in the same sphingolipid class with the closest number of carbon atoms in their acyl chains. During the concentration calculations, for the sphingolipids without their deuterated analogues as internal standards, Cer (d18:1-d7/20:1) or SM (d18:1-d9/22:1) was used as a common internal standard.

Gangliosides were detected using a Thermo Ultimate 3000 UHPLC system coupled to an LTQ-Orbitrap Velos Pro with high-mass-resolution detection (FWHM 60 000 at m/z 400) in a mass range of m/z 300–2000 and in the positive ion mode. The gangliosides were assigned based on comparisons of the measured accurate masses of gangliosides to their theoretically calculated masses based on an allowable mass error of 3 ppm and with the aid of standard substances of the gangliosides GM1, GM2 and GM3. The ion chromatograms of the detected gangliosides were extracted using their accurate masses within a mass window of 3 ppm, and the peak areas were integrated and used for relative quantification across the different samples.

### SIN-GFP assays

Vero cells (3 × 10^4^ cells per well) were seeded in 96-well plates. After overnight incubation, the cells (16 replicates) were treated with 10 µM GZ-161 or GZ-346. One hour later, the cells were infected with SIN-GFP (MOI = 5). The GFP levels were measured at 1, 3, 5, 7 and 9 hpi using an Infinite 200 M Plex plate reader (TECAN). For the measurement of GFP expression in individual SIN-GFP-infected cells, Vero cells (1.5 × 10^5^ cells per well) were seeded in 6-well plates. After overnight incubation, the cells were treated in triplicate with 10 µM GZ-161 or GZ-346. One hour later, the cells were infected with SIN-GFP (MOI = 5), and at 24 hpi, the cells were collected and stained with Live/Dead Cell Stain (Thermo Fisher, L34955). The GFP intensity of live cells (live/dead negative) were analysed by flow cytometry. Samples were collected using a Fortessa flow cytometer (BD Biosciences) and analysed with FlowJo software (TreeStar).

### Western blotting

The cells were washed twice with PBS and lysed in an RIPA buffer (Merck, R0278) supplemented with a protease inhibitor cocktail (Merck, P8340). The samples were sonicated twice for 5 s to fragment DNA and boiled for 5 min to denature proteins. The lysates were resolved by 12% SDS–PAGE (GenScript, ExpressPlus™ PAGE Gel, M01212) and subsequently transferred onto a nitrocellulose membrane (Thermo Fisher, iBlot™ 2 Transfer Stacks, nitrocellulose, IB23002). The membrane was blocked with 5% bovine serum albumin (Biological Industries, 03-010-1B) in PBS-0.05% Tween 20 and incubated overnight with primary antibody at 4°C. After washing, the membrane was incubated with IRDye 800CW goat anti-rabbit secondary antibody (LI-COR, 926–32 211) for 1 h at 20°C. The membrane was washed with PBS-0.05% Tween and developed using an Odyssey®-CLx imaging system (LI-COR). The primary antibodies included mouse anti-α-tubulin (1:1000, Merck, T6199, clone DM1A) and anti-Sindbis nsP2 rabbit polyclonal antiserum (nsp2–2, 1:3000, kindly provided by Charles M. Rice, Rockefeller University, USA).

### Quantitative (real-time) reverse transcription PCR (qRT-PCR)

Supernatants were collected, centrifuged in a tabletop centrifuge for 5 min at a maximum speed and stored at −80°C. RNA was extracted with a Qiagen viral RNA extraction kit according to the manufacturer’s instructions. The RNA loads in media were determined by qRT–PCR. Real-time RT–PCR was conducted with a SensiFAST™ Probe Lo-ROX One-Step Kit (Bioline, 78005) and analysed using a 7500 Real-Time PCR System (Applied Biosystems). The PFU equivalent per milliliter (PFUE/ml) values were calculated from a standard curve generated from virus stocks. The primers used to generate the data are listed in Supplementary Table 5.

### Mouse studies

Twenty-one-day-old C57BL/6J mice (both males and females) purchased from Envigo (Israel) were used in all *in vivo* experiments. The animal experiments were conducted in accordance with the guidelines of the Israel Institute for Biological Research (IIBR) animal experiments committee (protocol number: M45-17). The animals were provided water and food *ad libitum* and housed in light/dark cycles of 12 h.

C57BL/6 mice were infected i.p. with SVNI (15 PFUs/mouse). Beginning at 16 days of age (5 days preinfection) or 23 days of age (2 days postinfection), injections of GZ-161 were administered i.p. Compound GZ-161 was dissolved in 30 mM citrate buffer in normal saline (pH 5) to obtain a 1 mg/ml stock solution.

The mice received two i.p. administrations of α-GalCer (KRN7000, Funakoshi) at a dose of 2 µg/mouse on days 0 and 3 post infection.

The mice were weighed daily throughout the experiment, and the dosages were adjusted accordingly. The control mice received a similar volume of injection buffer without the active agent. The mice were assigned to different treatment groups in a randomized manner.

### RNA-seq

#### RNA purification

The mouse brains (*n* = 3–5 per group) were dissected into the right and left hemispheres, and one hemisphere was flash frozen in liquid nitrogen and stored at −80°C until use. RNA was isolated using an RNeasy mini kit (Qiagen, Hilden, Germany). The RNA quality was examined with an RNA High-Sensitivity Kit (Agilent Technologies), and the RNA integrity number (RIN) of each sample was determined. Samples with RIN ≥ 8.6 were sent to JP Sulzberger Columbia Genome Center (New York, NY, USA) for sequencing.

#### Library preparation and sequencing

Libraries were constructed using a TruSeq RNA library preparation kit (Illumina), and whole-transcriptome sequencing (total RNA-seq) was performed on the Illumina HiSeq platform. Over 30 million single 100-nt reads were generated per sample.

#### Bioinformatic analysis

mRNA expression was quantified by DESeq after normalization of the library size with Pipeline.^31^ Gene lists were created based on the following filtering criteria: absolute linear fold change ≥ 2.0 and FDR *P* ≤ 0.05. Heatmaps were generated using Partek® software (Partek, Inc., St. Louis, MO, USA).^32^ GO biological process enrichment analysis was performed with ENRICHER.^33-35^ The STRING protein–protein interaction^36^ tool was used to specify interactions between apoptosis-related genes.

### Brain cell isolation

The procedure was conducted as previously described.^37^ The mice (*n* = 3–6 per group) were anaesthetized with a combination of ketamine and xylazine (100 and 10 mg/kg, respectively) and then transcardially perfused with PBS. The brains were dissected, coarsely chopped and incubated for 20 min at 37°C in 1 ml of HBSS containing 2% BSA, 1 mg/ml collagenase D (Sigma) and 50 µg/ml DNase I (Sigma). The homogenates were then filtered through a 150 μm mesh, washed with cold flow cytometry buffer (2% FCS and 1 mM EDTA in PBS without Ca^2+^ or Mg^2+^) and centrifuged at 970*×g* at 4°C for 5 min. The cell pellet was resuspended in 3 ml of 40% Percoll and recentrifuged at 970*×g*, with no acceleration and braking, at room temperature for 15 min. The cell pellet was resuspended, passed through an 80 µm mesh, washed with 5 ml of flow cytometry buffer, centrifuged at 400*×g* at 4°C for 5 min, and then subjected to antibody labeling and flow cytometry.

### Flow cytometry

Approximately 10^6^ cells were stained with Aqua or violet Live/Dead Cell Stain (Thermo Fisher, L34955), blocked with an anti-CD16/32 FcγR antibody for 15 min and subsequently stained with fluorescently labeled antibodies for 30 min. The following antibodies from Bio-Legend (San Diego, CA, USA) were used: FITC or Super Bright 600 anti-CD45 (clone 30-F11), APC-efluor780 anti-CD3ε (clone 145-2C11), Alexa Fluor 700 anti-CD4 (clone RM4–5), APC anti-CD8 (clone 56–6.7), PE-Cyanine5 anti-CD19 (clone 1D3), APC anti-Ly6G (clone 1AB-Ly6G) and PE-Cyanine7 anti-CD11b (clone M1/70) antibodies. The PE anti-mouse CD1d-PBS57 tetramer (PBS-57 is a synthetic analogue of α-GalCer) was provided by the NIH Tetramer Core Facility at Emory University (Atlanta, GA, USA). All washing procedures were performed using flow buffer comprising PBS, 2% FBS and 0.05% NaN_3_. Samples were collected using a Fortessa flow cytometer (BD Biosciences) and analysed using FlowJo software (TreeStar). The gating strategy is shown in Supplementary Fig. 1.

### Enzyme-linked immunosorbent assay (ELISA)

The serum levels of anti-SVNI antibodies were determined by ELISA. The ELISA plates were coated with 4.5 × 10^7^ PFUs/ml inactivated SV in carbonate-bicarbonate buffer (C-3041; Sigma-Aldrich, St. Louis, MO, USA) and incubated overnight at 4°C. The plates were then washed three times with PBS-T (phosphate-buffered saline [PBS] containing 0.05% [vol/vol] Tween 20) and blocked for 1 h with PBS-2% bovine serum albumin (BSA)−0.05% Tween 20 at 37°C. After three washes, the samples were incubated with mouse serum diluted 1:100 in PBS containing 1% BSA for 1 h and then detected by alkaline phosphatase-anti-mouse immunoglobulin G (whole molecule) antibody (A-4312; Sigma). Values of at least twice the background signal (serum of uninfected mice) were considered positive (*n* = 4 for uninfected, *n* = 9 for SVNI, and *n* = 12 for SVNI + GZ-161).

### Statistical analysis

Statistical analyses were performed with two-tailed unpaired t tests or as indicated in the legends; the *P* values are indicated by asterisks in the figures as follows: *P < 0.05, **P < 0.01, ***P < 0.001 and ****P < 0.0001. Differences with *P* ≤ 0.05 were considered significant. The exact values of *n*, representing the number of mice in each experiment, are indicated in the figure legends. All measurement data are expressed as the means ± SEMs.

For the analysis of mouse survival, Kaplan–Meier survival curves were generated and analysed for statistical significance using GraphPad Prism 6.0 (log-rank [Mantel–Cox] test [conservative]).

All experimental measurements were obtained from distinct samples, and every sample was measured at least twice. The values presented were obtained from the distinct samples.

### Data availability

The accession codes for the RNA-seq datasets reported in this paper can be found in the GEO database GSE171912 (https://www.ncbi.nlm.nih.gov/geo/query/acc.cgi).

All data and materials used within this study will be made available, upon reasonable request, to research groups wishing to reproduce/confirm our results.

## Results

### SVNI infection induces alterations in sphingolipid levels early post infection

**Figure 1:**
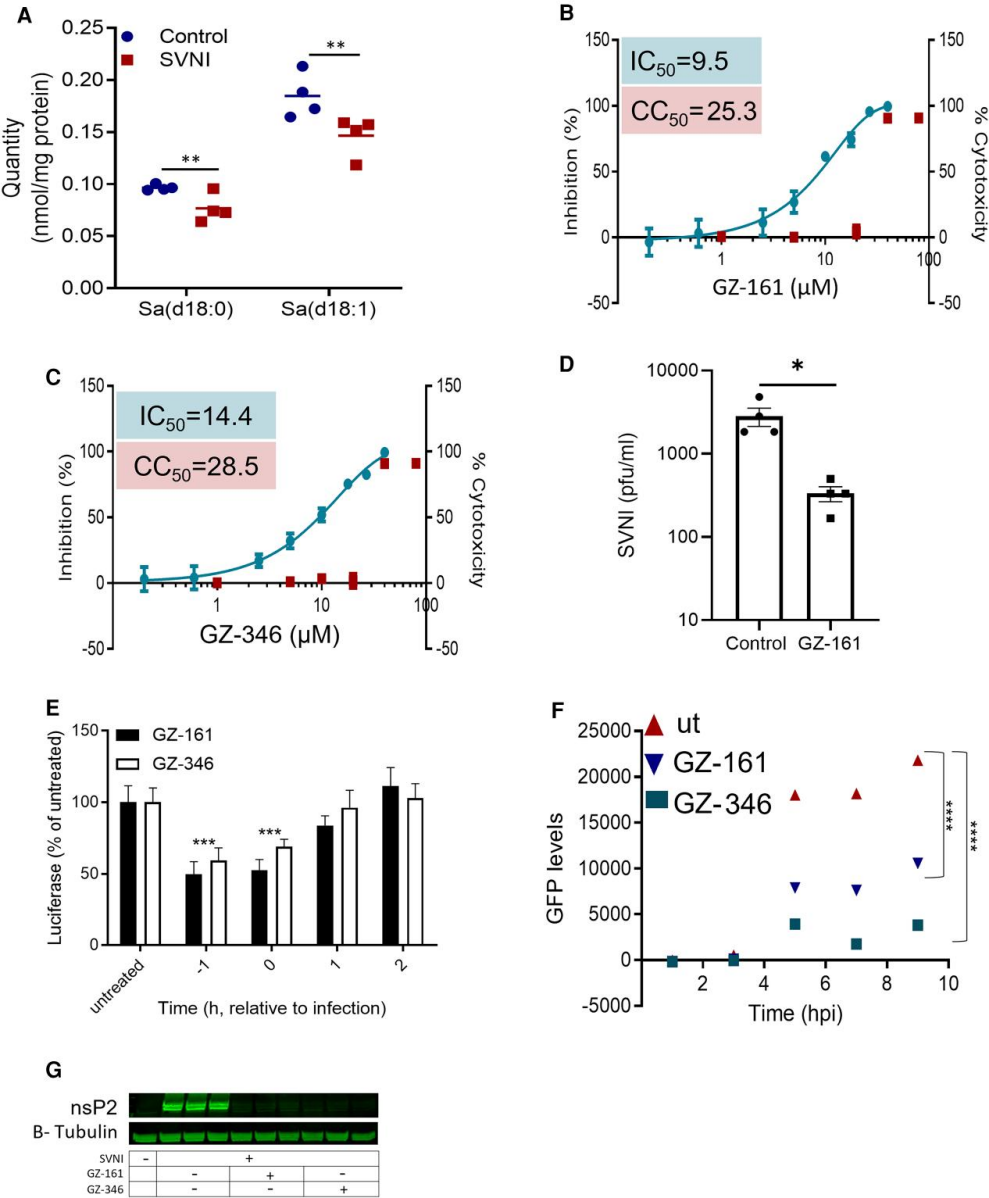
**Inhibition of SVNI by UGCG inhibitors.** (**A**) Reduced levels of Sa d18:0 and Sa d18:1 3 h after SVNI infection. Vero cells were infected with SVNI (MOI = 5), and the SL levels were quantified by UPLC-MRM/MS at 3 hpi (dataset in Supplementary Table 1). The data are the means ± SEMs of four biological replicates. Statistical analysis was performed by one-way ANOVA with Tukey’s *post hoc* test (α = 0.05). **P < 0.01. (B, C) Dose–response curves of GZ-161 (**B**) and GZ-346 (**C**) on inhibiting infection with recombinant neuroadapted SV expressing luciferase (i.e. TRNSV-Luc). Vero cells were treated with GZ-161 or GZ-346 (0.2–80 µM) and infected 1 h later with TRNSV-Luc (MOI = 0.01). The infected cells were lysed 24 h later, and the luciferase activities were measured. The viability of the uninfected cells was determined by an XTT assay. Measurements were obtained from distinct samples. The data are the means ± SEMs of quadruplicates. The IC50 values of GZ-161 and GZ-346 were 9.5 and 14.4 µM, respectively. (**D**) Reduced SVNI release after treatment with GZ-161. Vero cells were treated with GZ-161 (10 µM). One hour later, the cells were infected with SVNI (MOI = 5). Viral release into the media was measured by plaque forming unit (PFU) assay at 24 h post infection. The data are the means of four replicates ± SEMs. Each data point represents the PFUs/ml in a single well. The statistical analysis was performed using a two-tailed unpaired t test. * *P* < 0.05. (**E**) Time-of-drug-addition assay. Vero cells were treated with 10 µM GZ-161 or GZ-346 1 h prior to infection (−1), immediately postinfection (0) and at 1 hpi (1) or 2 hpi (2). The cells were infected with TRNSV-Luc (MOI = 0.01) on ice for 1 h after washing. The infected cells were lysed 24 h later, and the luciferase activity was measured. Measurements were obtained from distinct samples. The data are the means ± SEMs of 12 replicates. The statistical analysis was performed by two-way ANOVA with Dunnett’s *post hoc* test (α = 0.05). ***P < 0.001 versus the infected untreated group. (**F**) Inhibition of subgenomic GFP expression by UGCG inhibitors. Vero cells were treated with 10 µM GZ-161 or GZ-346 1 h prior to infection. The cells were infected with SIN-GFP (MOI = 5) on ice for 1 h after washing. The GFP levels were measured repeatedly in a microplate reader at the indicated time points after infection. The data are the means ± SEMs of 16 replicates. The statistical analysis was performed by one-way ANOVA with Dunnett’s *post hoc* test (α = 0.05). ****P < 0.0001 versus the infected untreated group. (**G**) Reduced levels of the SVNI nonstructural protein nsP2 in UGCG inhibitor-treated cells at 4 hpi as determined by Western blotting. Vero cells were infected with SVNI (MOI = 5) in the presence of GZ-161 (10 µM) or GZ-346 (10 µM) or were left untreated (UT). UGCG inhibitors were added to the media 1 h before infection. Tubulin protein was used as the loading control. Triplicates from distinct samples are presented. Uncropped blots are shown in Supplementary Fig. 7.

To evaluate the involvement of SLs in the SVNI life cycle, the SL levels in Vero cells 3 h postinfection (hpi) with SVNI were quantified by UPLC-MRM/MS (the dataset is provided in Supplementary Table 1). To ensure single-cycle infection, a high multiplicity of infection (MOI) of 5 was applied. SVNI infection reduced the levels of both sphinganine (Sa d18:0) and sphingosine (Sa d18:1) (Fig. 1A), suggesting activation of *de novo* biosynthesis of SLs by SVNI at early time points after infection. No increases in the levels of Cer, ceramide-1-phosphate (C1P), sphinganine-1-phosphate (d18:0), sphingosine-1-phosphate (S1P) (d18:1), lactosylceramide (LacCer), sphingomyelin (SM), or hexosylceramide (HexCer, GalCer + GlcCer) were detected (Supplementary Table 1). This result might imply that the end product of the increased *de novo* synthesis of SLs induced by SVNI is a more complex glycosphingolipid.

### Inhibition of SVNI by glucosylceramide synthase inhibitors

To examine the role of glycosphingolipid synthesis in the life cycle of SVNI, the antiviral activity (inhibitory effects of viral replication in cell culture) of UGCG inhibitors against SVNI was evaluated. Vero cells were incubated with serial dilutions of GZ-161 or GZ-346 1 h prior to infection with SVNI-expressing luciferase (TRNSV-Luc).^38^ Both GZ-161 and GZ-346 exhibited antiviral activity, with average IC50 values of 9.5 and 14.4 μM, respectively (Fig. 1B and C). No cytotoxicity was observed in similarly treated uninfected cultures across the dose range (half-maximal cytotoxic concentration (CC50) of 25.3 μM for GZ-161 and 28.5 μM for GZ-346). In addition to the reduction in the luciferase signal, a significant reduction in viral release at a GZ-161 concentration of 10 µM was observed in the plaque assay (Fig. 1D).

To confirm the inhibitory activity of GZ-161 and GZ-346 against UGCG, UPLC-MRM/MS analysis was performed. As expected, both inhibitors reduced the levels of all-chain HexCers (Supplementary Fig. 2, Supplementary Table 1) and increased the levels of long-chain Cers (C22, C24 and C26) (Supplementary Fig. 2, Supplementary Table 1). We then examined the antiviral activity of UGCG inhibitors in a mouse neural crest-derived cell line, Neuro 2A (N2a), to confirm that their antiviral activity was retained in cells of neuronal origin. GZ-161 and GZ-346 inhibited SVNI replication in both cell lines with inhibition percentages of ∼40–50% and ∼20–30% in Vero and N2a cells, respectively (Supplementary Fig. 3).

### UGCG inhibitors interfered with an early stage of the SVNI replication cycle

To determine which stage of the SVNI infection cycle is affected by UGCG inhibitors, a time-of-addition assay was performed. As shown in Fig. 1E, the most efficient inhibition was obtained when UGCG inhibitors were added before or at the time of infection (Fig. 1E). To further elucidate the mechanism through which UGCG inhibitors suppress the SVNI life cycle, SV encoding the green fluorescent protein (GFP) gene under the control of a subgenomic promoter (SIN-GFP)^30^ was used. Vero cells were incubated with 10 μM GZ-161 or GZ-346 for 1 h prior to infection with SIN-GFP (MOI = 5), and the GFP signal was measured at intervals of 2 h (Fig. 1F). Although GFP expression was highly elevated in the untreated cells, a robust reduction was observed in the GZ-161- and GZ-346-treated cells compared with the untreated cells (Fig. 1F). To determine whether the GFP level was reduced due to a decrease in the number of GFP-expressing cells or to overall impairment of GFP expression in all cells, a flow cytometric analysis was performed (Supplementary Fig. 4). Both GZ-161 and GZ-346 significantly reduced the number of GFP-expressing cells at 24 hpi from ∼90% in the untreated cell cultures to ∼60% and ∼30% in the GZ-161- and GZ-346-treated cell cultures, respectively. The similarity of the median levels of GFP in treated and untreated cells (Supplementary Fig. 4) implies that GZ-161 and GZ-346 do not interfere with viral protein synthesis. To determine whether GZ-161 and GZ-346 interfere with viral genome replication or exert their effects at an earlier stage in the replication cycle, the level of the nonstructural protein nsP2 was determined by Western blotting. nsP2 is part of the RNA polymerase complex and is synthesized from viral genomic RNA prior to replication of the viral genome. GZ-161 and GZ-346 significantly reduced the level of the nsP2 protein in infected cells at 4 hpi (Fig. 1G).

### GZ-161 extends the survival of SVNI-infected mice

**Figure 2:**
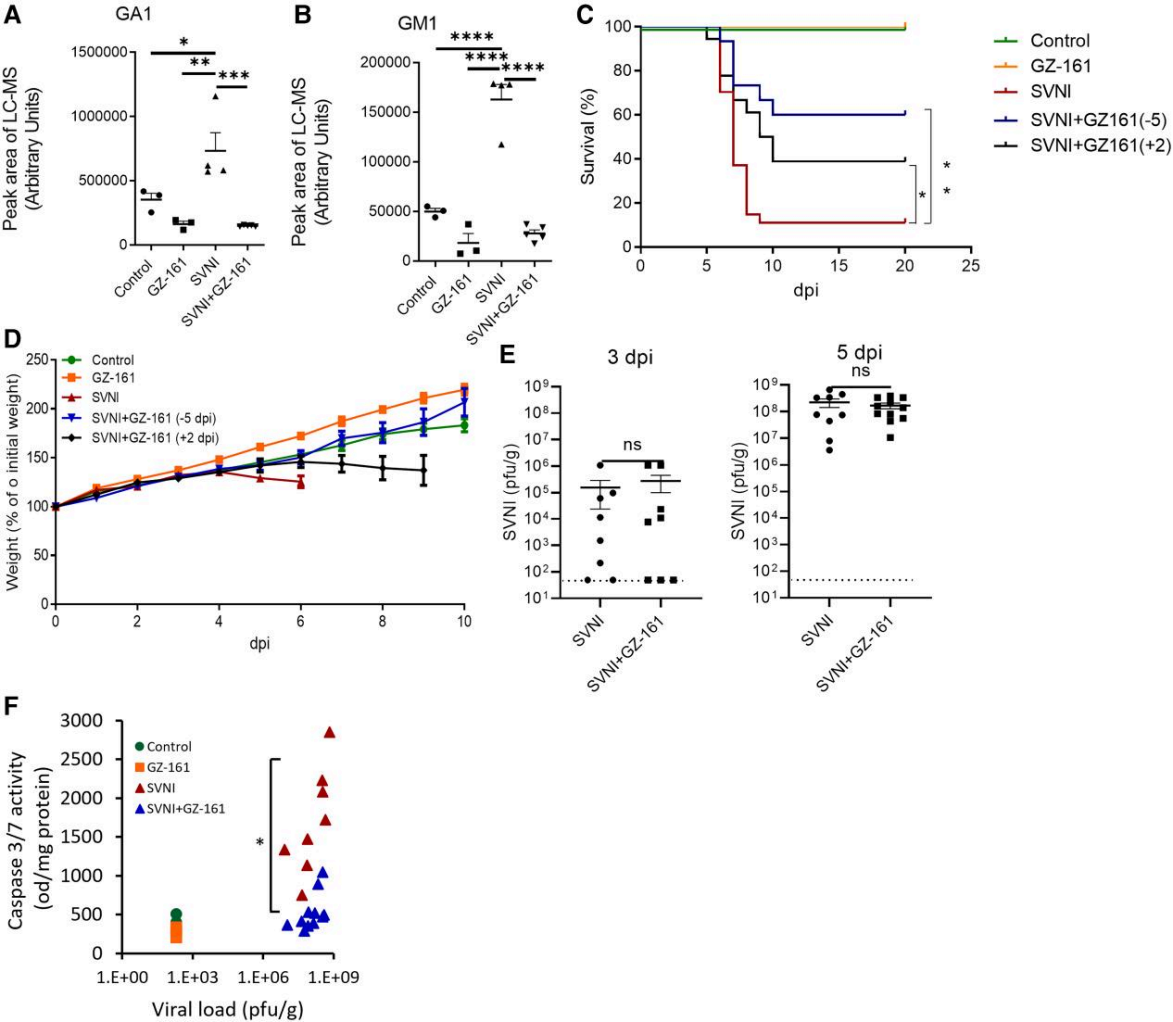
**GZ-161 extends the survival of SVNI-infected mice.** (**A, B**) SVNI infection induces elevation in the GA1 (**A**) and GM1 (**B**) levels in murine serum. The mice were either infected with SVNI (15 PFUs, administered i.p.) or not infected (control, *n* = 3; 2 females, 1 male) and were either left untreated (SVNI, *n* = 3; 2 females, 1 male) or treated with GZ-161 (20 mg/kg/day, i.p., beginning on day 5 preinfection; GZ-161, *n* = 4, 2 females, 2 males; SVNI + GZ-161, *n* = 5, 2 females, 3 males). The ganglioside levels in serum samples obtained at day 5 postinfection were analyzed by LC–MS. The LC–MS peak area was divided by milligrams of protein in the sample for calibration. GA1, asialo GM1. The statistical analysis was performed by one-way ANOVA followed by a Tukey’s multiple comparison test. The *P* values are indicated by asterisks as follows: *P < 0.05, **P < 0.01, ***P < 0.001 and ****P < 0.0001. Differences with a *P* value of 0.05 or less were considered significant. Graphs were generated using GraphPad Prism software version 8.4.3. (**C**) Kaplan–Meier survival curves of SVNI-infected mice (15 PFUs, administered i.p.). Mice were either left untreated (SVNI, *n* = 27; 15 females, 12 males) or treated with GZ-161 (20 mg/kg/day, i.p., beginning on day 5 preinfection [SVNI + GZ-161 (−5), *n* = 15; 8 females, 7 males] or day 2 postinfection [SVNI + GZ-161 (+2), *n* = 18; 10 females, 8 males]. The control mice were left uninfected (*n* = 6; 3 females, 3 males). Comparisons of the Kaplan–Meier survival curves by the log-rank test indicated a significant decrease in the mortality of GZ-161-treated mice compared with the SVNI mice. *P < 0.05; **P < 0.01. (**D**) Body weight (% of infection day 0) of C57BL/6 mice untreated (control, *n* = 5; 2 females, 3 males) or treated with GZ-161 (20 mg/kg per day, *n* = 8; 4 females, 4 males) beginning on day 5 preinfection [SVNI + GZ-161 (−5), *n* = 15; 8 females, 7 males] or day 2 postinfection [SVNI + GZ-161 (+2), *n* = 6; 3 females, 3 males]. The mice were left uninfected or infected with a lethal dose (15 PFUs) of SVNI (SVNI, *n* = 15; 7 females, 8 males) at 21 days of age. The results are the means ± SEs. (**E**) The SVNI viral load in brain homogenates was measured by a PFU assay at 3 and 5 dpi. Similar viral loads in the brains of SVNI and SVNI + GZ-161 mice were detected. GZ-161 (20 mg/kg/day, i.p.) was administered beginning on day 5 preinfection. The results are the means ± SEMs (*n* = 8 (4 females, 4 males) for each group at 3 dpi, at 5 dpi: *n* = 9 (4 females, 5 males) for SVNI, *n* = 11 (6 females, 5 males) for SVNI + GZ-161). The statistical analysis was performed by a two-tailed unpaired t test. ns, not significant. The dotted line reflects the limit of detection (LOD) (**F**) Activity of caspase-3/7 versus the viral load in brain homogenates at 5 dpi. Each dot represents the value found for the brain (*n* = 3, control and GZ-161; *n* = 8, SVNI; and *n* = 11, SVNI + GZ-161). The statistical analysis was performed using the Kruskal–Wallis test followed by Dunnett’s *post hoc* test for pairwise comparisons. A significant reduction in caspase-3 activity was detected in SVNI + GZ-161 cells compared with SVNI cells (*P < 0.05).

We subsequently examined whether the alteration in the levels of SLs induced by SVNI was also observed *in vivo* and whether treatment with GZ-161 could affect it. Based on the *in vitro* data, which suggested increases in more complex GSLs, the levels of gangliosides and more complex GSLs were measured. The mice were treated with GZ-161 (20 mg/kg/day, intraperitoneally [i.p.]) and infected with a lethal dose of SVNI. The ganglioside levels in serum were measured 5 days postinfection (dpi). The levels of GA1 (C:16) and GM1 (C:16:0) were significantly increased in the sera of SVNI-infected mice, and treatment with GZ-161 significantly prevented this elevation (Fig. 2A and B). No significant differences in the levels of GA2, GM2 and GM3 were detected between SVNI-infected and naïve mice (Supplementary Table 1).

We then evaluated whether GZ-161 exerts protective effects in SVNI-infected mice and found that GZ-161 significantly protected pretreated infected mice from SVNI-induced death (Fig. 2C). Virtually, all SVNI-infected mice died within 9 days postinfection [∼90% mortality, with a median survival time (T50) of 7 days], whereas 60% of SVNI-infected animals treated with GZ-161 survived (Fig. 2C, with an undefined median survival time). Postponing the treatment to the second day after infection preserved some level of protection in SVNI-infected mice (Fig. 2C); 40% of mice receiving postinfection treatment with GZ-161 survived (with a median survival time of 9.5 days) (Fig. 2C).

Notably, whereas control mice infected with SVNI displayed typical signs of morbidity, i.e. weight loss and manifestations of viral CNS infection (Fig. 2D), such as hind limb paralysis, the signs of disease were ameliorated in the GZ-161-treated mice that survived (Fig. 2D). The inhibition of SVNI replication *in vitro* suggested that the beneficial effect of SVNI *in vivo* might be due to a reduced viral load. Thus, we examined whether the viral load in mice was reduced after treatment with GZ-16. After peripheral inoculation, the primary target cells of SVNI are neurons.^39^ Indeed, no viral load was detected in peripheral organs such as the liver and spleen of SVNI-infected mice at 5 dpi (LOD = 400 PFUs/g tissue). Unexpectedly, the analysis of brain tissue at 5 dpi revealed a nonsignificant slight reduction (∼30%) in the viral load in the GZ-161-treated mice compared with the untreated mice (Fig. 2E). The average viral loads in the control and GZ-161-treated mice were 2.2e8 PFUs/g and 1.7e7 PFUs/g, respectively (Fig. 2E). In addition, no significant reduction in viral load was observed at 3 dpi, the earliest time point of viral entry into the brain, when the average viral loads in the control and GZ-161-treated mice were 2.93e5 PFUs/g and 1.7e5 PFUs/g, respectively (Fig. 2E). Although no overt difference in viral load was observed after treatment with GZ-161 (Fig. 2E), significant reductions in the levels of the apoptosis markers caspase 3/7 were observed in the brains of the treated mice. Only 2 of 11 GZ-161-treated mice showed caspase 3/7 activity above the control levels at 5 dpi (Fig. 2F). The significant reduction in caspase 3/7 activity found in GZ-161-treated brains, even though these brains had a high viral load similar to that found in the untreated brains, suggests that GZ-161 has an additional mechanism of action *in vivo* in addition to the reduction in viral load that is responsible for the reduced brain pathology after SVNI infection.

### Treatment with GZ-161 decreases the detrimental immune response in the brain after SVNI infection

To shed light on the mechanism underlying the acquisition of increased resistance to viral infection in the GZ-161-treated mouse brains, we performed high-throughput RNA sequencing (RNA-seq) of RNA isolated from the hemibrains of mice belonging to the control, GZ-161, SVNI and SVNI + GZ-161 groups at 5 dpi.

**Figure 3:**
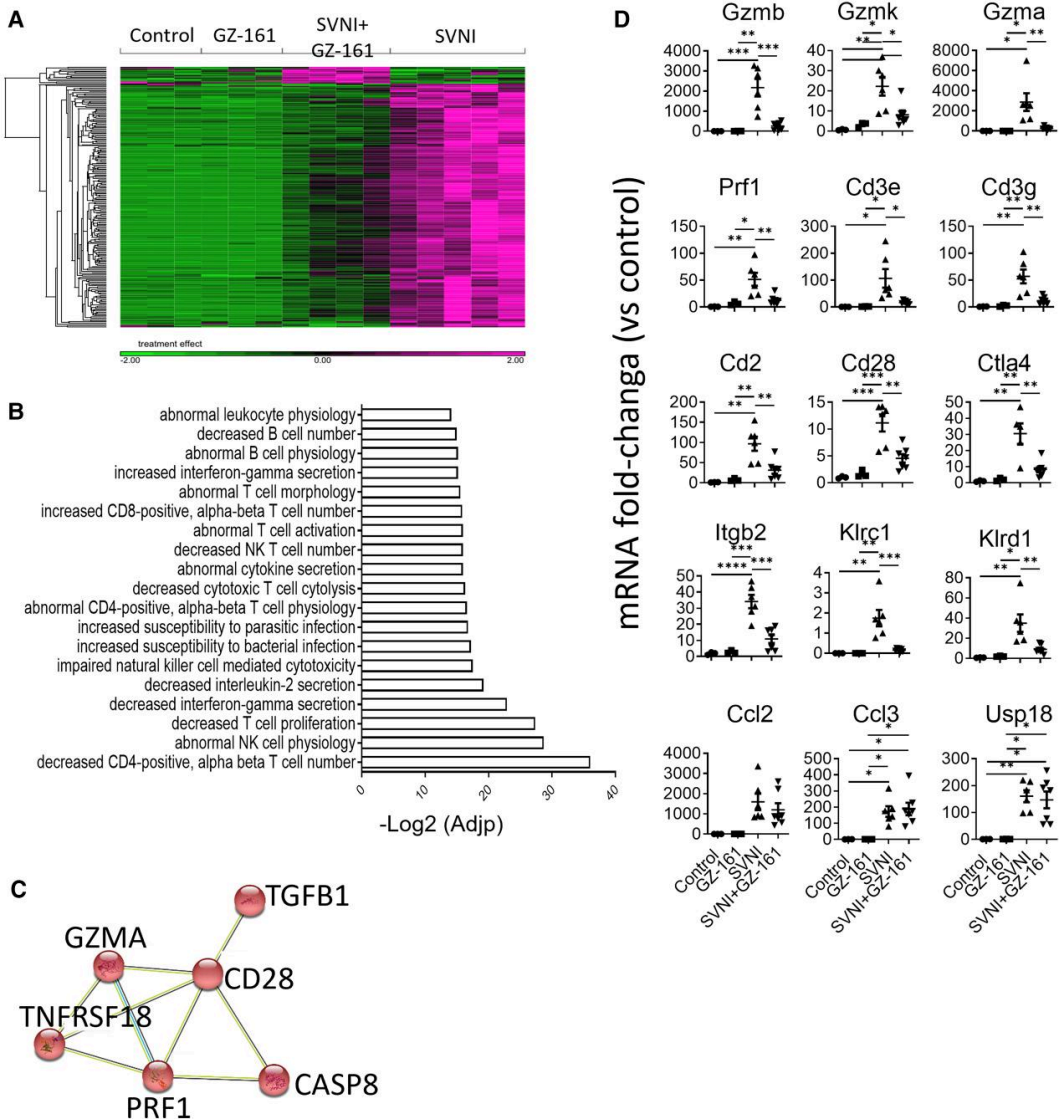
**Treatment with GZ-161 decreases immune response pathways in the brain after SVNI infection.** (**A**) Heatmap of RNA-seq results comparing the brains of the mice in the control, GZ-161, SVNI, and SVNI + GZ-161 groups. Lists of **216** DEGs between the SVNI and SVNI + GZ-161 groups are shown. The data sets are provided in Supplementary Table 4. Each column represents an individual mouse [*n* = 3 (2 females, 1 male), control and GZ-161]; *n* = 5 [3 females, 2 males, SVNI; *n* = 4 (2 females, 2 males), SVNI + GZ-161]. (**B**) ENRICHR biological process enrichment analysis of the 216 DEGs was performed. The top-ranked biological processes are presented. (**C**) GZ-161 treatment decreased the levels of apoptosis-related genes. DEGs were analyzed using the STRING database to create a protein–protein interaction (PPI) network. The analysis was performed using default parameters. The red color indicates genes involved in apoptotic process (GO: 0006915 Apoptotic process, *P*[FDR] = 6.62e-06. (D) qPCR of selected genes in cortical homogenates from mice of the control, GZ-161, SVNI and SVNI + GZ-161 groups at 5 dpi. The results are presented as fold changes versus the control values and are expressed as the means ± SEMs. Each data point represents the mRNA fold-change in a single mouse brain. The CT values were normalized to the HPRT levels. The statistical analysis was performed by one-way ANOVA with Tukey’s *post hoc* test (α = 0.05). *P < 0.05; **P < 0.01; ***P < 0.001; ****P < 0.0001; *n* = 3, control and GZ-161; *n* = 4–6, SVNI; and *n* = 7, SVNI + GZ-161.

The gene expression in the brains of the GZ-161 group was similar to that in the brains of the control group (no significant differentially expressed transcripts [fold change ≥ 2, FDR *P* ≤ 0.05]). A total of 1700 upregulated genes and 119 downregulated genes were found in the mice in the SVNI group compared with those in the control group. GZ-161 treatment reduced the overall number of differentially expressed genes (DEGs) upon SVNI infection, consistent with the observed increase in resistance to SVNI-induced encephalitis; 612 upregulated genes and only 18 downregulated genes were found in the SVNI + GZ-161 group compared with the control group (see Fig. 3A, Supplementary Fig. 5, and Supplementary Table 2 for a complete list of the DEGs) (absolute fold change ≥ 2, FDR *P* ≤ 0.05).

To identify the enriched pathways in the brains of SVNI + GZ-161-treated mice that might be responsible for the protection observed in the GZ-161-treated mice, we compared the DEGs in the brains of mice belonging to the SVNI group compared with the SVNI + GZ-161 group. A total of 216 genes were differentially expressed in the mice of the SVNI group compared with the mice belonging to the SVNI + GZ-161 group (Fig. 3A, Supplementary Table 2 for a complete list of the DEGs) (absolute fold change ≥ 2, FDR *P* ≤ 0.05). These genes were next subjected to gene ontology (GO) enrichment analysis with ENRICHER (Fig. 3B, Supplementary Table 3), which indicated that the T-cell-, B-cell-, natural killer (NK) cell- and natural killer T (NKT) cell-related pathways were significantly suppressed in the mouse brains of the SVNI + GZ-161 group compared with the SVNI group (Fig. 3B, Supplementary Table 3). Moreover, a significant (*P*[FDR] = 6.62e-06) decrease in the apoptotic pathway was detected after treatment with GZ-161 (Fig. 3C), which was in line with the observed reduction in caspase-3 activity.

To confirm the RNA-seq results, a quantitative PCR (qPCR) analysis of selected genes (Fig. 3D) was performed. GZ-161 treatment significantly reduced the mRNA expression levels of the apoptosis-induced genes granzyme B (*Gzmb*), granzyme K (*Gzmk*), granzyme A (*Gzma*), and perforin 1 (*Prf1*). Cytotoxic T lymphocytes (CTLs), NK cells and iNKT cells utilize perforin and granzymes to kill infected cells,^40,41^ suggesting that a reduction in these types of cells may explain the reduced caspase-3/7 activity detected after GZ-161 treatment. GZ-161 treatment significantly reduced the mRNA levels of the T lymphocyte and NKT cell markers *Cd3e*, *Cd3g*, *Cd2*, *Cd28* and *Ctla4* and the level of the panleukocyte marker integrin beta chain-2 (*Itgb2*, also called CD18) (Fig. 3D). Additionally, the mRNA levels of killer cell lectin-like receptor C1 (*Klrc1*, also called CD159) and *Klrd1* (also called CD94), markers of NK/iNKT cells, were significantly reduced after treatment with GZ-161. Although distinct reductions in adaptive immunity-related genes were observed, no differences were observed in the mRNA levels of the more general inflammatory cytokines C-C motif chemokine ligand 2 (*Ccl2*) and C-C motif chemokine ligand 3 (*Ccl3*) or ubiquitin-specific peptidase 18 (*Usp18*), which mediates the regulation of the inflammatory response to type 1 interferon (Fig. 3D).

**Figure 4:**
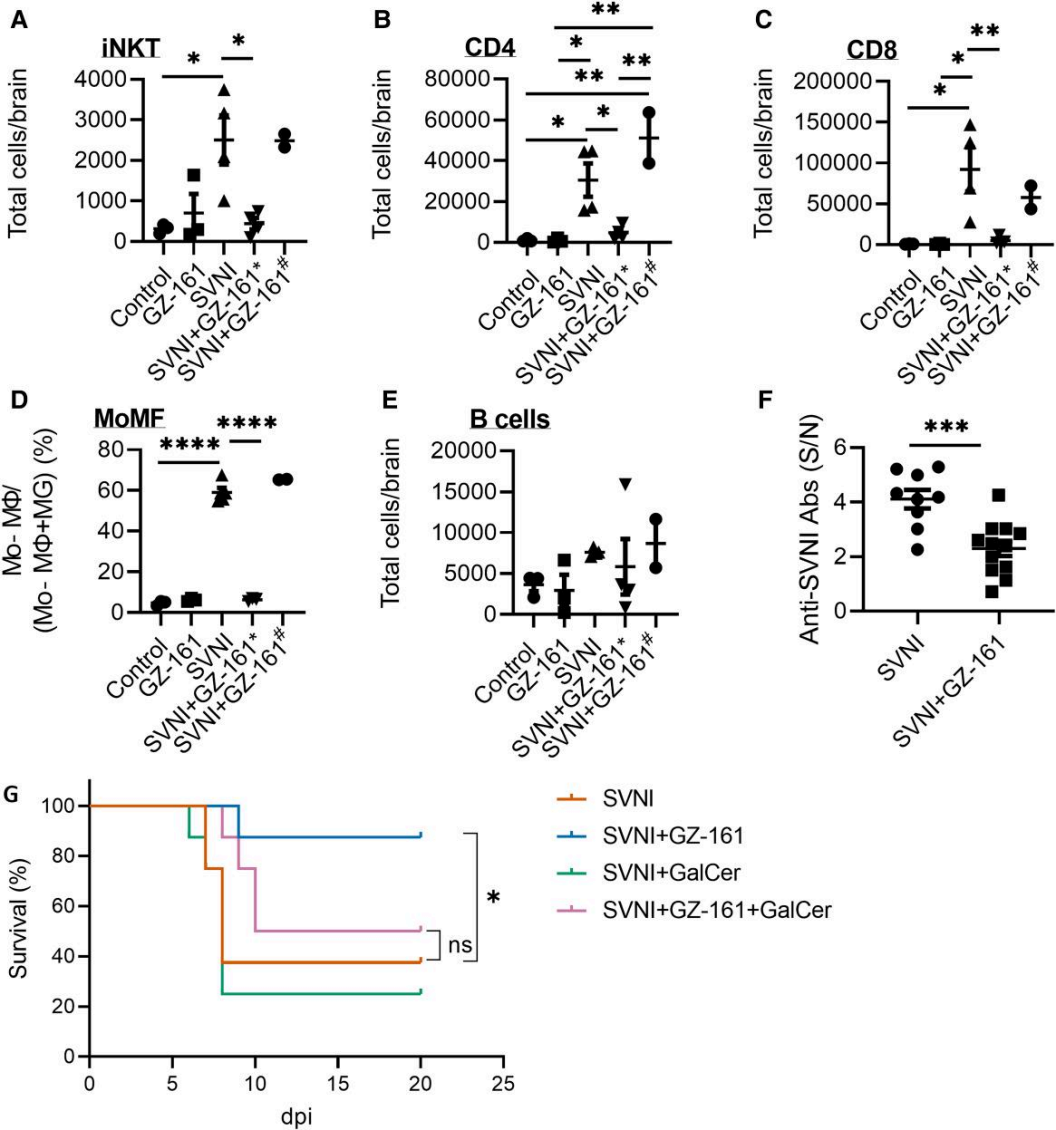
**Treatment with GZ-161 decreases the number of immune cells in the brain after SVNI infection.** (**A–E**) Flow cytometry analysis of iNKT, CD4 + and CD8+ T cells, monocyte-derived macrophages (Mo-MΦs), and B cells in the brains of control (*n* = 3, 1 female, 2 males), GZ-161 (*n* = 3, 1 female, 2 males), SVNI (*n* = 4, 2 females, 2 males), and SVNI + GZ-161 (*n* = 6, 3 females, 3 males) mice at 5 dpi. The SVNI + GZ-161 group was divided into two subgroups according to the weight loss experienced by the treated mice. Four mice did not show signs of disease at 5 dpi (SVNI + GZ-161*), and two mice lost weight (SVNI + GZ-161^#^). CD4^+^ T cells (CD45 ^+^ CD3ε^+^CD4^+^), CD8^+^ T cells (CD45 ^+^ CD3ε^+^CD8^+^), iNKT cells (CD45 ^+^ CD3ε^+^CD1d-PBS57+), B cells (CD45 ^+^ CD19^+^), Mo-MΦs (Ly6G^−^CD11b^hi^ CD45^hi^), and microglia (MG) (Ly6G^−^CD11b^hi^ CD45^int^). The percentage of Mo-MΦs within the total myeloid lineage cell population [Mo-MΦ/(Mo-MΦ +MG)] is shown. The data are presented as the total cell numbers per brain. The statistical analysis was performed by one-way ANOVA with Tukey’s *post hoc* test (α = 0.05). *P < 0.05; **P < 0.01; ****P < 0.0001. (**F**) Serum levels of anti-SVNI antibodies (total) in C57BL/6 mice left untreated (SVNI, *n* = 9, 4 females, 5 males) or treated with GZ-161 (20 mg/kg per day beginning on day 5 preinfection) (SVNI + GZ-161, *n* = 12, 6 females, 6 males) and infected with SVNI (15 PFUs, administered i.p.). Each data point represents the cell number in a single mouse brain. The anti-SVNI antibody levels were measured by ELISA at 5–6 dpi. The signal/noise (S/*N*) ratios in the SVNI and SVNI + GZ-161 groups were determined by dividing the mean absorbance of the test sera by the mean absorbance of the sera from control and GZ-161 mice, respectively. The results are presented as the means ± SEMs. The statistical analysis was performed using a two-tailed unpaired t test. ***P < 0.001. (**G**) α-GalCer abolished the therapeutic effect of GZ-161. Survival rate of C57BL/6 mice infected with SVNI (15 PFUs) at 21 days of age. The mice were not treated (SVNI), treated with GZ-161 (SVNI + GZ-161, 20 mg/kg per day, i.p.) starting from 13 days of age, treated with α-GalCer (SVNI + GalCer, 2 µg/mouse, twice at days 0 and 3 post infection, i.p.) or treated with both GZ-161 and α-GalCer. Comparisons of Kaplan–Meier survival curves by the log-rank test indicated a significant increase in the survival of the SVNI + GZ-161 mice compared with the SVNI mice. *P < 0.05 and no significant (ns) difference between SVNI and SVNI + GZ-161+ α-GalCer. *n* = 8 mice/group (4 females, 4 males).

Flow cytometry was then performed to distinguish among T, B, NK, iNKT and monocyte-derived macrophage (Mo-MΦ) cells (Fig. 4A–E). Infection with SVNI induced the infiltration of CD4^+^ and CD8^+^ T cells, Mo-MΦ, and iNKT cells (Fig. 4A–D). Treatment with GZ-161 significantly reduced the number of infiltrated cells in the animals that did not lose weight at 5 dpi (indicative of animals that benefit from GZ-161 treatment; Fig. 3D, marked with *) but had no effect on the animals that lost weight (Fig. 4A–E, marked with #). These results suggest that GZ-161 treatment reduced the detrimental immune response to SVNI.

SVNI infection also increased the number of B cells in the brain (albeit not significantly; Fig. 4E). Thus, to further explore the B-cell response to SVNI upon GZ-161 treatment, the serum levels of anti-SVNI antibodies (total) were measured at 5 dpi. GZ-161 reduced the SVNI antibody titers compared with those in the SVNI group, consistent with a less robust adaptive immune response (Fig. 4F).

To evaluate whether GZ-161 exerts protective effects in SVNI-infected mice by reducing iNKT activation, the mice were treated with GZ-161 together with glycolipid α-galactosylceramide (α-GalCer), which is a potent and specific iNKT activator. The activation of iNKT cells by α-GalCer eliminated the protective effect of GZ-161 (Fig. 4G). This finding supports the notion that the beneficial mechanism of action of GZ-161 *in vivo* is due to reduced GSL-induced immune response pathways.

### The antiviral activity of UGCG inhibitors is not specific to SVNI

Subsequently, we examined whether GZ-161 has therapeutic potential against a neurotropic virus from a different genus—West Nile virus (WNV). WNV is a neurotropic flavivirus that is the leading cause of arboviral encephalitis worldwide.^3^ Vero cells were incubated with 10 μM GZ-161 or GZ-346 1 h prior to WNV infection. Supernatants were harvested 24 hpi and analysed by qPCR. We observed a 60% reduction in the viral RNA titer in the supernatant of cells treated with GZ-161 or GZ-346 compared with that of cells treated with vehicle (DMSO, untreated) (Supplementary Fig. 6A). We then evaluated whether GZ-161 can protect WNV-infected mice. C57BL/6 mice were treated with GZ-161 starting 5 days prior to infection (20 mg/kg/day, i.p.) and infected with a lethal dose of WNV. GZ-161 delayed the day of disease onset (weight loss) and increased (albeit nonsignificantly) the median survival time from 9.5 to 14 days (Supplementary Fig. 6B–D). Although the effect of GZ-161 on WNV-infected mice was less substantial than that on SVNI-infected mice, the effect was notable and suggests a key role of the glycosphingolipid synthesis pathway in viral CNS infection.

## Discussion

The present study shows that SVNI induce an alteration in the levels of SLs early after infection both *in vitro* and *in vivo*, and this alteration appears to play a role in SVNI replication, as indicated by the finding that UGCG inhibitors reduced viral replication *in vitro*. The precise role of SLs in the viral life cycle has not been fully resolved. Our data imply that UGCG inhibitors inhibit the SVNI infection cycle after viral attachment and before translation of genome-encoded proteins. SLs play a significant role in endocytosis and thus might play a major role in viral entry into the cell.^42^ Previous studies have shown that knockout of the UGCG-encoding gene impairs the entry of the influenza virus by endocytosis,^7^ which is consistent with our data showing that UGCG inhibitors interfere with an early stage of the SVNI replication cycle.

Although UGCG inhibitors significantly inhibit SVNI replication, the ratio of IC50 to CC50 for the antiviral activity of UGCG inhibitors *in vitro* is high. The IC50 of the antiviral activity of the tested UGCG inhibitor was found to be in the low µM range, whereas the IC50 values for the inhibition of UGCG were in the nM range.^43^ The relatively high concentration needed for the antiviral effect could indicate that (i) the antiviral effect requires inhibition of UGCG activity by more than 50% and (ii) antiviral activity is caused by the inhibition of other pathways, which require higher concentrations. The findings that similar inhibition was observed with two UGCG inhibitors with different structures (GZ-161 and GZ-346) and that other UGCG inhibitors and that the knockout of UGCG inhibited other viruses renders the possibility of an ‘off-target’ effect less likely. Moreover, the lack of significantly reduced viral levels *in vivo* in response to GZ-161 treatment demonstrates that a high dose of GZ-161 is needed for the inhibition of SVNI replication.

Our *in vivo* experiments showed that the GZ-161-treated mice had lower caspase 3/7 activity in the brain despite a similarly high viral load (Fig. 2F). This result implies that a high SVNI viral load alone does not lead to neuronal cell death. Increases in mortality and caspase 3/7 activity were observed only when there was also a significant induction of the immune response. This finding suggests that the cause of brain tissue death is the inflammation/immune response that is triggered by viral infection rather than the viral levels themselves. This conclusion is consistent with other studies showing that neuronal death in SVNI infection is mediated by the immune system rather than a direct consequence of viral infection and associated with the entry and differentiation of pathogenic T cells in the nervous system.^1,37^

Although the viral load in the brains of both GZ-161-treated and untreated mice were similarly high, the immune response level was significantly reduced in the GZ-161-treated animals (Figs 3 and 4). Therefore, the lowered immune response in the GZ-161-treated mice cannot be explained by a reduced viral load and must be explained by some other impact of GZ-161 treatment. Because GZ-161 treatment has been shown to prevent the elevation of serum SL levels induced by SVNI infection (Fig. 2A and B), we hypothesize that an increase in the SL levels plays a role in immune system activation in response to SVNI infection. In fact, elevation of the SL levels is well known to induce an immune response, specifically a type I IFN response.^44-48^

Viral-induced elevation of the SL levels has been shown to be associated with a number of viruses, such as Zika virus,^46^ hepatitis C virus (HCV),^49^ human cytomegalovirus (HCMV),^50^ dengue virus,^49^ influenza virus^51,52^ and SARS-CoV-2.^9^ It is therefore plausible that the immune system has evolutionarily developed the identification of high SL levels as a marker for viral infection to trigger the antiviral response.

Because UGCG catalyzes the first step in glucosphingolipid synthesis, its inhibition results in reduced amounts of not only GlcCer but also other glycosphingolipids.^27-29^ Membrane microdomains termed lipid rafts formed by glycosphingolipids on cellular membranes play important roles in T and B-cell activation.^53-55^ In addition, several lines of evidence imply that GlcCer-based glycosphingolipids and gangliosides might be lipid antigens relevant to invariant natural killer T (iNKT) cell development^56^ and activation.^57-61^

Our data suggest that GZ-161 alter NKT cell activation and reduce the immune response in SVNI mice. Activated NKT cells can provide maturation signals to downstream cells, including dendritic cells (DCs), NK cells, and lymphocytes. Although viruses do not contain lipid antigens, NKT cells have been implicated in antiviral responses.^62,63^ iNKT cells provide innate and adaptive help for B cells, and mice, which lack type I and II NKT cells, exhibit diminished B-cell responses during influenza and vaccinia virus infection.^64,65^

The enrichment of iNKT cells during viral CNS infection is detrimental in mice, as observed in Theiler’s murine encephalomyelitis virus (TMEV).^66^ Thus, impeding iNKT activation by reducing excessive inflammatory responses in some types of acute viral encephalitis might be beneficial and should be further explored. Our finding is consistent with the results from previous studies suggesting a role for pathogenic Th17 cells and CD4^+^ and CD8^+^ T cells in fatal SVNI encephalitis.^67-69^ Thus, the protection observed in GZ-161-treated mice could result directly from the suppression of cytotoxic and detrimental immune response pathways activated by SVNI and not from a reduction in viral replication.

The absence of a viral load in other organs discounts the assumption that the beneficial effect of GZ-61 is due to reduced viral titers in organs other than the brain. This conclusion is consistent with previous studies showing that the primary target of SVNI is the brain and that the ability of SVNI to spread to the CNS is the cause of fatal disease.^39^ In addition, while GZ-161 significantly improved the survival rate of the mice, GZ-346, which cannot penetrate the brain (35), had no effect when administered beginning 2 days post-SVNI infection (data not shown), which suggested that the inhibition of UGCG in the brain is necessary for therapeutic effects.

Although treatment with GZ-161 was also effective when its administration was started after viral exposure, a more significant effect was observed when its administration began prior to exposure. Therefore, both therapeutic treatment and prophylactic treatment are worth considering for populations at high risk.

The development of successful antiviral treatments remains a challenge.^70^ Historically, drug research has mainly focused on targeting viral components due to the perceived specificity of such an approach.^71^ However, host mechanisms, such as those presented in this study, can also be explored as antiviral targets. Although side effects may be of particular concern for such treatments, this approach has distinct advantages, such as creating a high barrier to resistance, providing broad coverage of different genotypes/serotypes, possibly even multiple viruses, and expanding the list of potential targets for a drug when druggable viral targets are limited.^72^

Another advantage of targeting host proteins is the availability of many approved drugs against host proteins, allowing drug repurposing. Drugs targeting SL-metabolizing enzymes are currently in use and are constantly being developed for the treatment of lysosomal storage diseases and other disorders in which alterations in the SL levels are involved in disease pathology,^73-75^ which allows potential repurposing of these already approved drugs as antivirals.

Taken together, our results indicate that GSLs play a major role in alpha virus-induced brain pathology. Alterations in the SL levels are induced by SVNI and play a role in viral replication. In addition, alterations in the SL levels are likely to contribute to detrimental host immune responses in the brain. The inhibition of UGCG synthesis may be a beneficial approach for the treatment of viral infection of the CNS.

## Supplementary Material

fcad086_Supplementary_Data

## Abbreviations

CNS =: central nervous system
Cer =: ceramide
C1P =: ceramide-1-phosphate
CerS =: ceramide synthase
CTLs =: cytotoxic T lymphocytes
DCs =: dendritic cells
UGCG =: with UDP-glucose ceramide glucosyltransferase
GlcCer =: glucosylceramide
GSLs =: glycosphingolipids
HexCer =: hexosylceramide
IFN =: interferon
LacCer =: lactosylceramide
NKT =: natural killer T
NK =: natural killer
SV =: Sindbis virus
SVNI =: neuroinvasive Sindbis virus
Sa =: sphinganine
SLs =: sphingolipids
SM =: sphingomyelin
So =: sphingosine
S1P =: sphingosine-1-phosphate
