# Supplementary material for: Antiviral activity of glucosylceramide synthase inhibitors in alphavirus infection of the central nervous system
Source: Brain Commun. 2023 Mar 25;5(3):fcad086. doi: 10.1093/braincomms/fcad086 (PMC10165247; doi:10.1093/braincomms/fcad086)
Supplement: fcad086_Supplementary_Data [file fcad086_supplementary_data.zip › Supplementary Figures.docx]

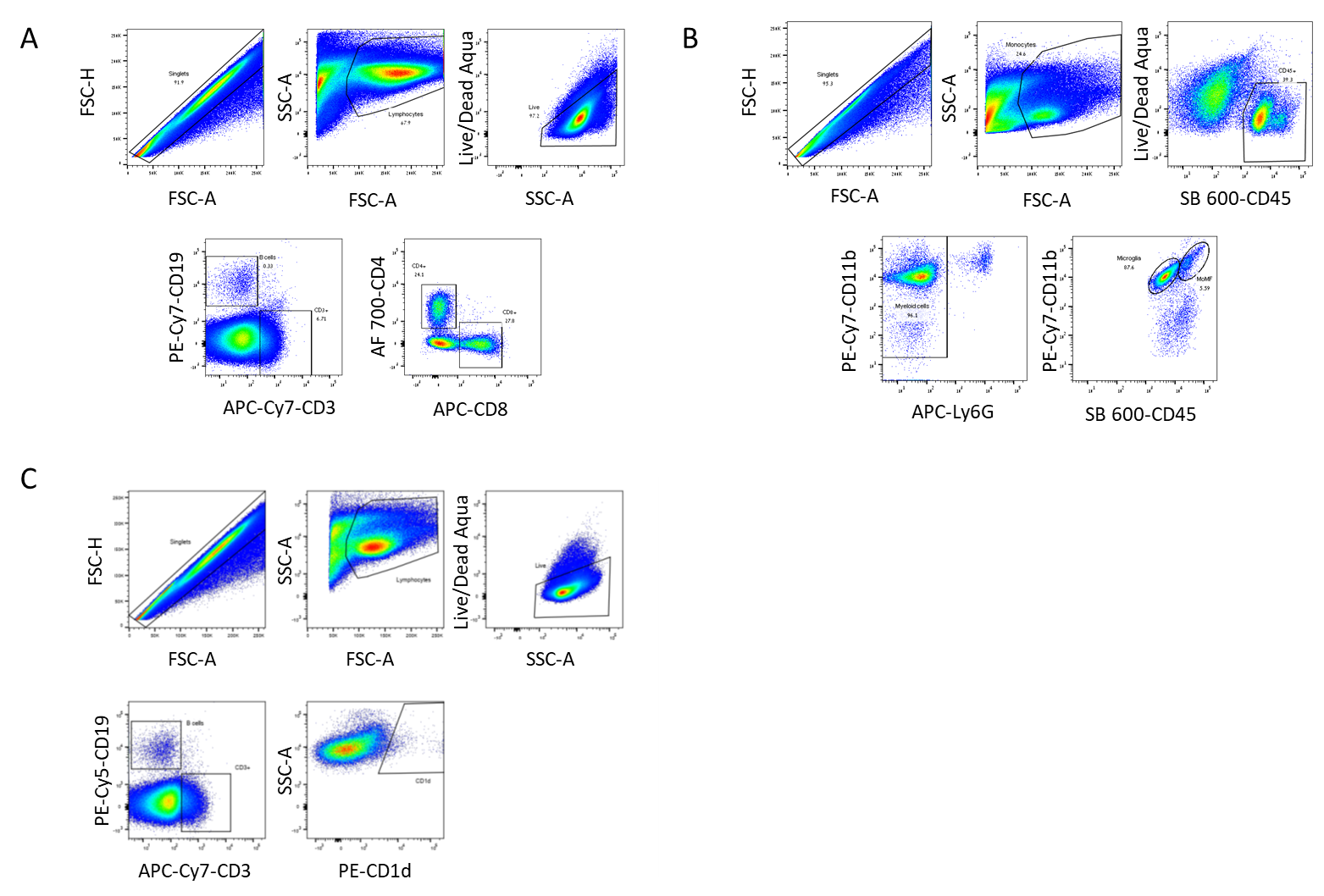


**Supplementary Fig. 1. Gating strategy for flow analysis**. (A) B cells, CD4^+^ T cells, and CD8^+^ T cells. (B) Microglia and monocyte-derived macrophages (MoMFs). (C) iNKT cells (CD1d^+^). Because no clear population of CD3^+^ cells was observed, the possibility that some of the CD8^+^ cells are dendritic cells cannot be excluded.

**
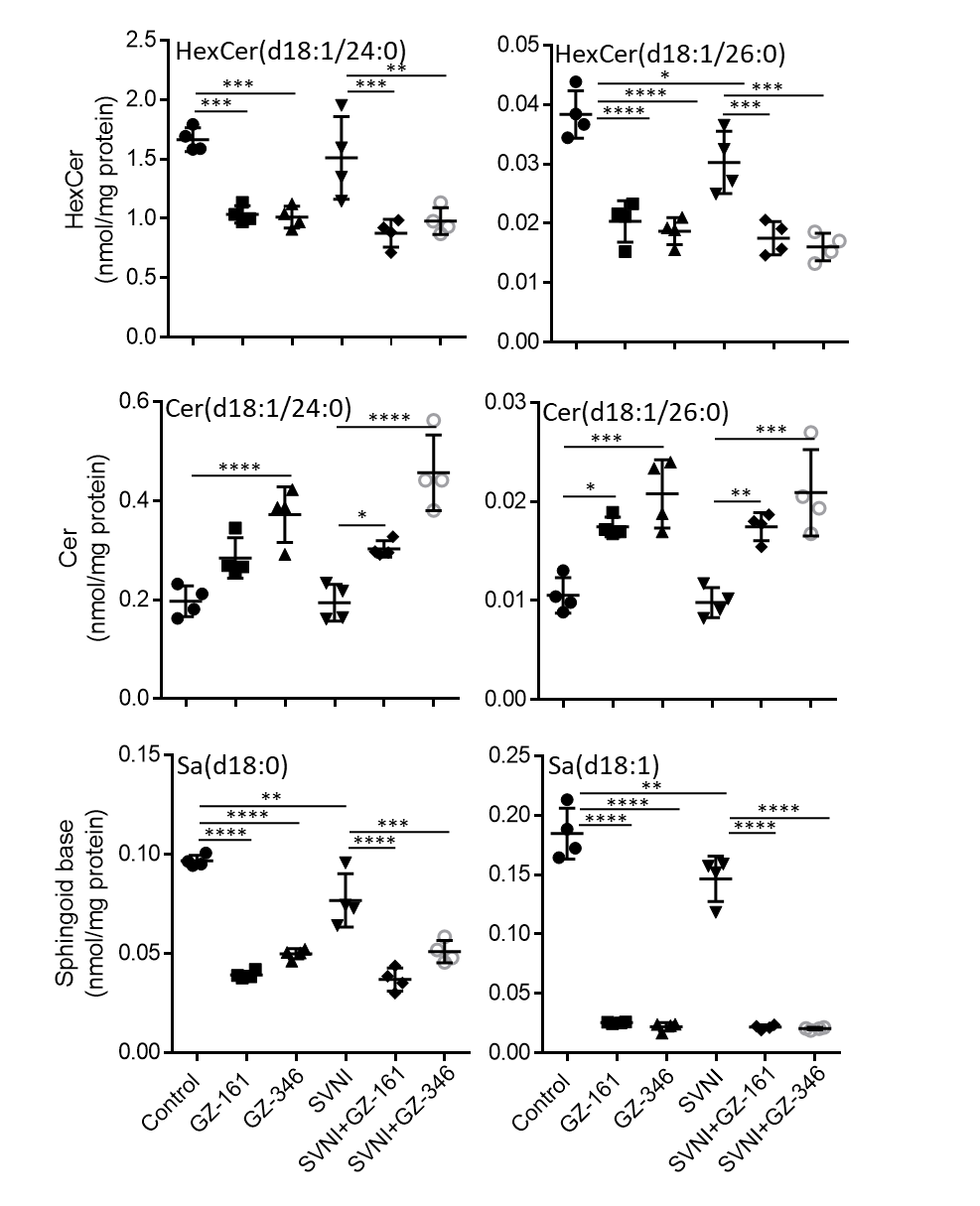
**

**Supplementary Fig. 2. Effect of GZ-161 and GZ-346 on the Cer and HexCer** **levels at 3 h post-SVNI infection.** The full dataset is provided in Supplementary Table 1. Vero cells were treated with 10 µM GZ-161 or GZ-346. The cells were infected 1 h later with SVNI (MOI = 5). At 3 hpi, the SL levels were quantified by UPLC-MRM/MS. n=4 for each group. The statistical analysis was performed by one-way ANOVA with Tukey’s post hoc test (α=0.05). *p<0.05; **p<0.01; ***p<0.001; ****p<0.0001.

**Supplementary Fig. 3.** GCS inhibitors inhibit SVNI replication in both Vero and N2a cells. Vero and N2a cells (3 × 10^4^ cells per well) were seeded in 96-well plates. After overnight incubation, the cells were treated with GZ-161 or GZ-346 (10 µM). The cells were infected 1 h later with TRNSV-Luc (MOI = 0.01). The infected cells were lysed 24 h later, and the luciferase activities were measured. Measurements were obtained from distinct samples. The data are the means of six replicates ± SEMs. The statistical analysis was performed by two-way ANOVA with Dunnett’s post hoc test (α=0.05). ***p<0.001 versus the infected untreated group.

**
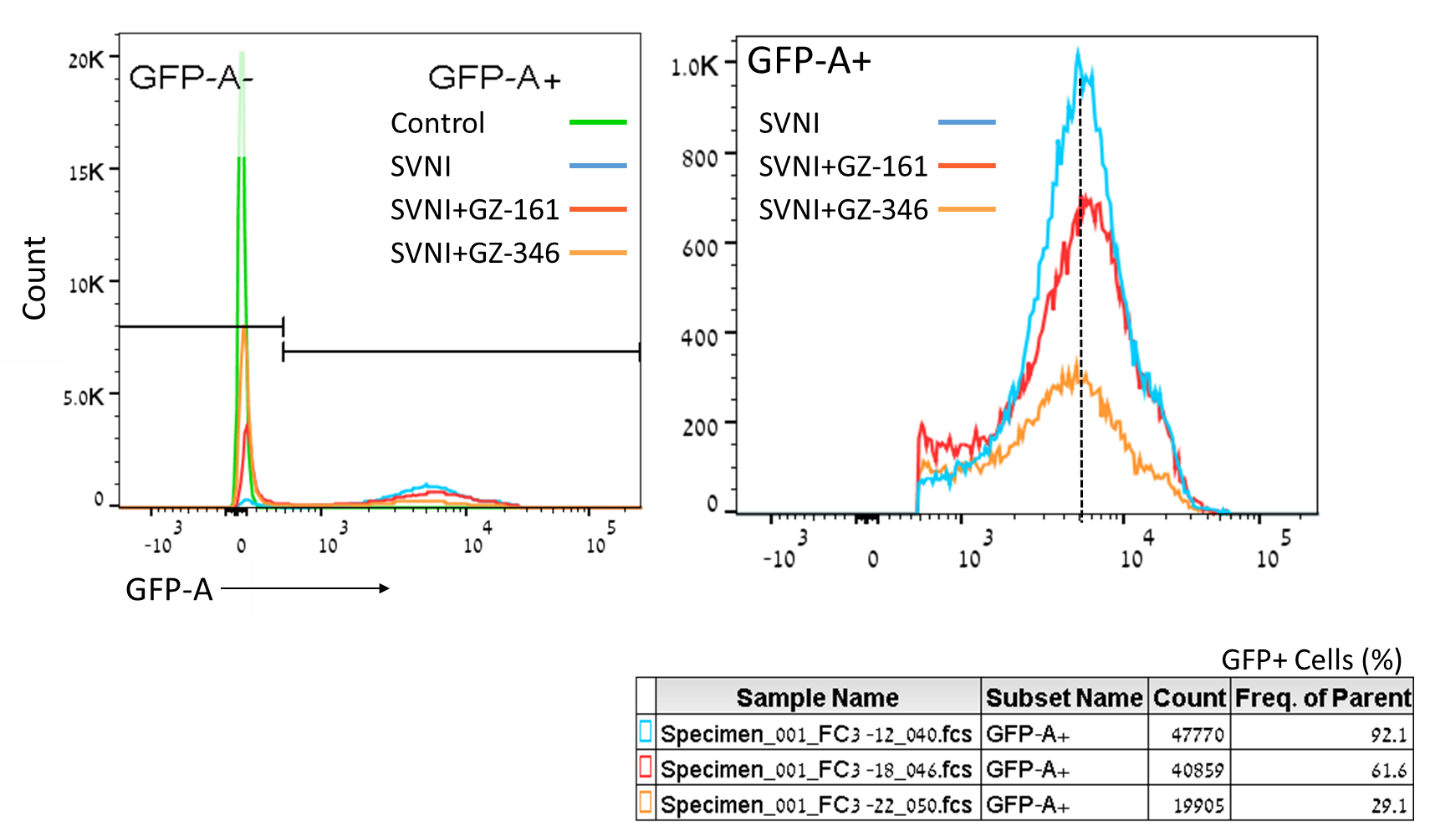
**

**Supplementary Fig. 4. GCS reduces the percentage of infected cells and does not affect the level of GFP transcription per cell.** Vero cells were treated with 10 µM GZ-161 or GZ-346 1 h prior to infection. The cells were infected with SIN-GFP (MOI = 5) on ice for 1 h after washing. At 24 hpi, the cells were analyzed by flow cytometry. The data are representative of triplicates.

**
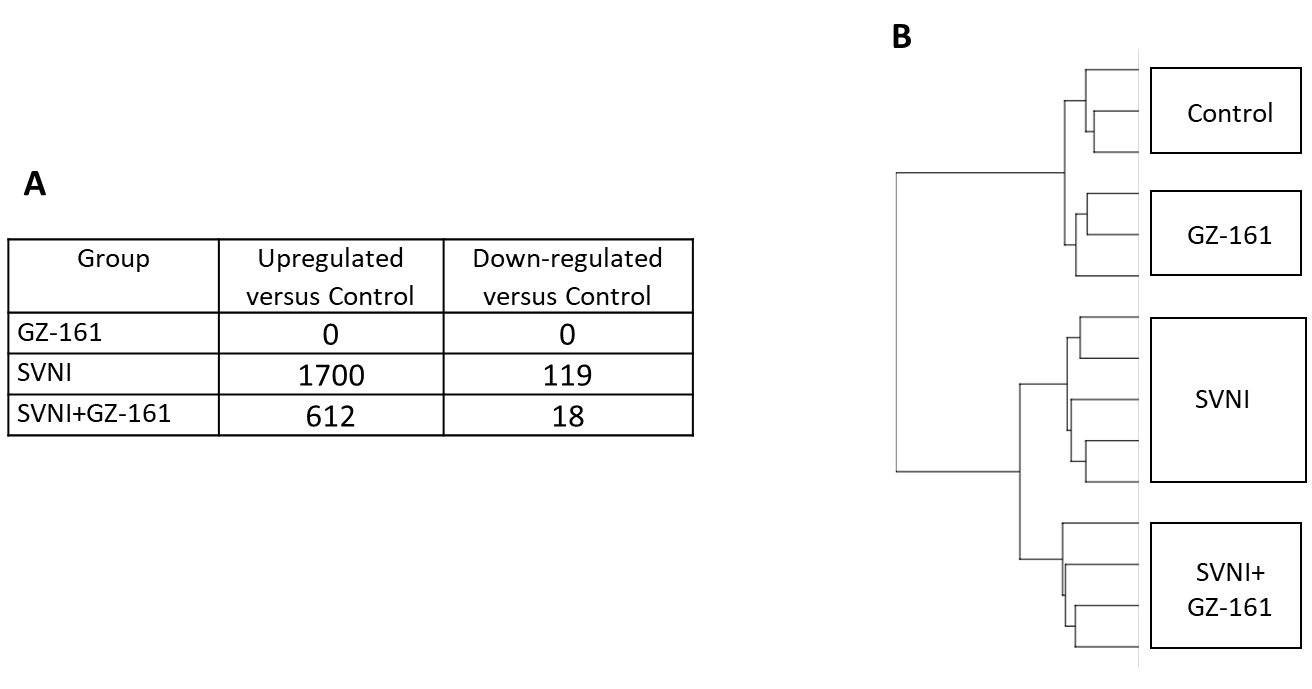
**

**Supplementary Fig. 5. Overview of the RNA-seq analysis.** **(A)** Number of up- and downregulated genes in the experimental group compared with the control group. **(B)** A cluster analysis of the experimental samples revealed the homogeneity of the replicates. (Control, n=3; GZ-161, n=3; SVNI, n=5; and SVNI+GZ-161, n=4).


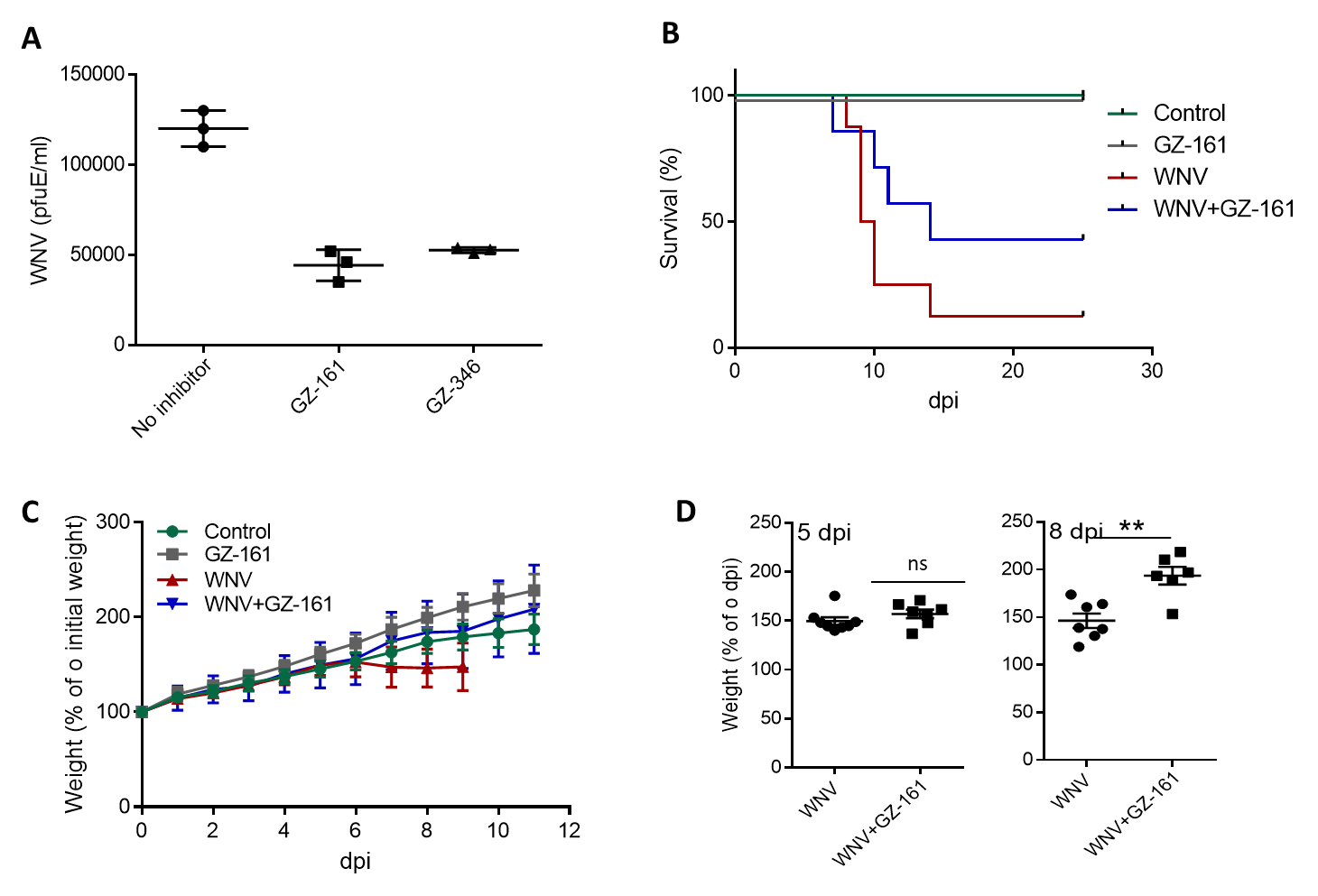


**Supplementary Fig. 6. Inhibition of WNV infection by GCS inhibitors. (A)** Inhibition of WNV infection by GCS inhibitors *in vitro.* Vero cells were treated with GZ-161 or GZ-346 (10 µM). One hour later, the cells were infected with WNV diluted in Eagle’s minimal essential medium (MOI = 0.1). The bar graph shows the effect of GZ-161 and GZ-346 on viral release. At 24 hpi, the viral release into the medium was measured by real-time PCR. The data are the means of triplicates ± SEMs. The statistical analysis was performed using a two-tailed unpaired t test. ***p<0.001 versus the infected untreated group. **(B)** Kaplan–Meier survival curves of WNV-infected mice (30 PFUs, administered i.p.). The mice were infected and not treated (WNV, n=8) or treated with GZ-161 (20 mg/kg/day, i.p.) beginning on day 5 preinfection (WNV+GZ-161, n=8). The control mice were not infected (n=8) or treated with GZ-161 (GZ-161, n= 8). **(C)** Body weight (% of infection day 0) of C57BL/6 mice left untreated (control, n=5, 2 females, 3 males) or treated with GZ-161 (20 mg/kg per day, n=8, 4 females, 4 males) beginning on day 5 preinfection (WNV+GZ-161 (-5), n=7, 4 females, 3 males) or left uninfected or infected with a lethal dose (30 PFUs) of WNV (WNV, n=8, 4 females, 4 males) at 21 days of age. The results are the means±SEMs. Only 4 mice in the WNV group survived at 10 dpi; thus, the last point on the graph is 9 dpi. **(D)** Body weights of WNV- and WNV+GZ-161-treated mice at 5 and 8 dpi indicating improved morbidity of the GZ-161-treated mice at 8 dpi. The statistical analysis was performed using a two-tailed unpaired t test. ns, not significant; **p<0.01.


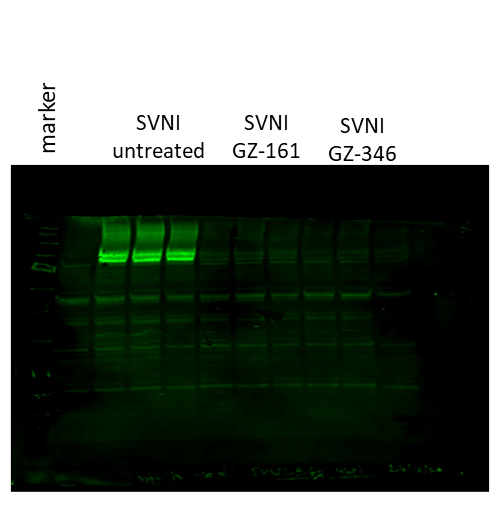


**Supplementary Fig. 7.** Uncropped blot of Figure 1G.
