# Supplementary material for: Antiviral activity of glucosylceramide synthase inhibitors in alphavirus infection of the central nervous system
Source: Brain Commun. 2023 Mar 25;5(3):fcad086. doi: 10.1093/braincomms/fcad086 (PMC10165247; doi:10.1093/braincomms/fcad086)
Supplement: fcad086_Supplementary_Data [file fcad086_supplementary_data.zip › Supplementary Table 5.docx]

| Gene name | Forward primer | Reverse primer |
| --- | --- | --- |
| *Hprt* | TGCTCGAGATGTCATGAAGG | AATCCAGCAGGTCAGCAAAG |
| *Gzmb* | CCACTCTCGACCCTACATGG | GGCCCCCAAAGTGACATTTATT |
| *Gzmk* | TGGCTGGCGTTTATATGTCTTC | GCTGCGGTACTGGATGGAC |
| *Gzma* | CTCCGTGGTGGAAAGGACTC | AGAGGTGATGCCTCGCAAAA |
| *Prf1* | CTGCCACTCGGTCAGAATG | CGGAGGGTAGTCACATCCAT |
| *Cd3e* | GTGCCTCTCCAGATTTCCCC | AGAGAGGGGGCTGGTGTATT |
| *Cd3g* | AGTGGCTTAAAGACGGGAGC | CCTCGAGGGTCTTTGGCATT |
| *Cd2* | TTCCTGGGTAGCTTCTTTCTGC | TTGGGGATGTTCAGGGTGATG |
| *Cd28* | CTATCAGCCCCAGTTTCGCTC | CGGAACGTCACTGTTTCGTTG |
| *Ctla4* | AGTGGGCTTCCTAGATTACCC | GTCCCGTGTCAACAGCTCTC |
| *Itgb2* | CAGGAATGCACCAAGTACAAAGT | CCTGGTCCAGTGAAGTTCAGC |
| *Klrc1* | GCCCCTGCAAAGATACCGAA | TCTGTGGGTTCTAGTCATTGAGG |
| *Klrd1* | CAAGTGGGTTGGGCATCAGT | AAGAAGGCTGGAATTCTGCGA |
| *Ccl2* | CCACAACCACCTCAAGCACT | AGGCATCACAGTCCGAGTCA |
| *Ccl3* | TTTTGAAACCAGCAGCCTTT | CTCAAGCCCCTGCTCTACAC |
| *Usp18* | CAGGAGTCCCTGATTTGCGT | GGGCTGGACGAAACATCTCA |

**Supplementary Table 5. Primers used for polymerase chain reaction.**

RNA was isolated using an RNeasy mini kit (Qiagen, Hilden, Germany). RNA (1 μg) was reverse-transcribed using the qScript cDNA Synthesis Kit (Quanta, 95047). Real-time PCR was conducted with PerfeCTa SYBR Green FastMix Low ROX (Quanta, 95074) and analyzed with the 7500 Real Time PCR System (Applied Biosystems). The relative amounts of mRNA were calculated from the cycle threshold (Ct) values using HPRT for normalization.
