# Supplementary material for: Antiviral activity of glucosylceramide synthase inhibitors in alphavirus infection of the central nervous system
Source: Brain Commun. 2023 Mar 25;5(3):fcad086. doi: 10.1093/braincomms/fcad086 (PMC10165247; doi:10.1093/braincomms/fcad086)
Supplement: fcad086_Supplementary_Data [file fcad086_supplementary_data.zip › Supplementary_Table_Legends_.docx]

**Supplementary data**

**Supplementary Table 1. Complete mass spectrometry data**

**Supplementary Table 2. Complete list of the DEGs**

RNA-seq data were obtained from the brains of control (n=3), GZ-161 (n=3), SVNI (n=5), and SVNI+GZ-161 (n=4) group mice at 5 dpi. Gene lists were created by filtering with the following criteria: absolute fold change≥2 and FDR p≤0.05.

Note: The raw read counts from the RNA-seq experiments can be found in the Gene Expression Omnibus (GEO; accession number GSE171912).

**Supplementary Table 3. List of significantly enriched biological processes.** An ENRICHR biological process enrichment analysis of the 216 DEGs was performed. The processes and genes involved are presented.

**Supplementary Table 4. Dataset used to construct the heatmap shown in Fig. 3.**

**Supplementary Table 5. Primers used for polymerase chain reaction.**
